# Medication Errors and Type 2 Diabetes Management: A Qualitative Exploration of Physicians' Perceptions, Experiences and Expectations From Quetta City, Pakistan

**DOI:** 10.3389/fmed.2022.846530

**Published:** 2022-03-28

**Authors:** Muhammad Kashif Habib, Muhammad Naeem Khan, Abdul Sadiq, Qaiser Iqbal, Abdul Raziq, Nafees Ahmad, Zaffar Iqbal, Sajjad Haider, Muhammad Anwar, Fazal ur Rehman Khilji, Fahad Saleem, Amer Hayat Khan

**Affiliations:** ^1^Faculty of Pharmacy & Health Sciences, University of Baluchistan, Quetta, Pakistan; ^2^Post Graduate Medical Institute, Bolan Medical Complex Hospital, Quetta, Pakistan; ^3^Department of Biochemistry, Jhalawan Medical College Khuzdar, Khuzdar, Pakistan; ^4^Department of Statistics, University of Baluchistan, Quetta, Pakistan; ^5^Health Department, Government of Balochistan, Quetta, Pakistan; ^6^School of Pharmaceutical Sciences, Universiti Sains Malaysia, Gelugor, Malaysia

**Keywords:** medication errors, diabetes mellitus, physicians, perceptions, experiences, expectations

## Abstract

**Background:**

Type 2 Diabetes-related medication errors are frequently reported from the hospitals and consequently are of major concern. However, such reports are insufficient when developing healthcare settings are pursued in literature. Keeping this inadequacy in mind, we therefore aimed to explore physicians' perceptions, experiences and expectations of medication errors when managing patients with Type 2 Diabetes Mellitus.

**Methods:**

A qualitative design was adopted. By using a semi-structured interview guide through the phenomenology-based approach, in-depth, face-to-face interviews were conducted. Physicians practicing at the medicine ward of Sandeman Provincial Hospital, Quetta, were purposively approached for the study. All interviews were audio-taped, transcribed verbatim, and were then analyzed for thematic contents by the standard content analysis framework.

**Results:**

Although the saturation was reached at the 13th interview, we conducted additional two interviews to ensure the saturation. Fifteen physicians were interviewed, and thematic content analysis revealed six themes and nine subthemes. Mixed conceptualization and characterization of medication errors were identified. Medication errors were encountered by all physicians however poor understanding of the system, deficiency of logistics and materials were rated as barriers in reporting medication errors. Among contributors of medication errors, physicians themselves as well as dispensing and patient-related factors were identified. Physicians suggested targeted training sessions on medication error-related guidelines and reporting system. Parallel, establishment of an independent unit, involving the pharmacists, and strict supervision of paramedics to minimize medication errors was also acknowledged during data analysis.

**Conclusion:**

With a longer life expectancy and a trend of growing population, the incidences of medication errors are also expected to increase. Our study highlighted prescribing, dispensing and administration phases as contributing factors of medication errors. Although, physicians had poor understanding of medication errors and reporting system, they believed getting insights on guidelines and reporting system is essential. A review of admission and discharge reconciliation must be prioritized and a culture of teamwork, communication and learning from mistakes is needed.

## Introduction

Diabetes being one of aglobal public health concern is approaching epidemic proportions. It is a serious burden to the healthcare systems that adversely affect the socio-economic development of nations (1). According to the International Diabetes Federation (IDF), 451 million adults had diabetes worldwide in 2017. Additionally, the IDF also estimated a projected increase of 693 million by the year 2045 provided no intervention is offered or adopted (2). While the past decades have seen significant progress in promoting population health and extending life expectancy, diabetes still has the second biggest negative effect on reducing global health adjusted life expectancy worldwide (3). Within this context, Type 2 Diabetes Mellitus (T2DM) is the most common form of diabetes accounting for around 90% of all global diabetes cases (4). Type 2 Diabetes Mellitus is characterized by persistent hyperglycemia, insulin resistance and reduced insulin levels in the body and is correlated to sedentary lifestyle, obesity, environmental, and genetic factors (5). The global prevalence of T2DM is also on the rise and it is predicted to be doubled in next decade (6). This increasing prevalence of T2DM has significant social, financial and development implications especially in low- and middle-income countries (7).

Together with T2DM, parallel development of comorbid conditions deteriorates the quality of life of diabetic patients (8). The circumstances are further complicated by medication errors (MEs) which are frequently encountered by both healthcare professionals and the patients (9, 10). Defined as “*any preventable event that may cause or lead to inappropriate medication use or patient harm while the medication is in the control of the healthcare professional, patient, or consumer*” (11), literature does report that MEs errors contribute to high number of morbidities, mortalities, and increased complications (12). Also, MEs are responsible for undesirable health consequences in patients that often result in increased hospitalization (13, 14). Correlating, Wilmer and colleagues identified multiple risk factors associated with MEs including comorbidities, polypharmacy, and the use of specific drugs (anti-cancer and antithrombotics etc.). The authors also commented that although several guidelines advise to use prespecified risk factors while managing ME, most of these known risk factors are insufficiently grounded on empirical evidence (15). Therefore, identifying factors related to MEs and interventions to resolve the complications are of prime importance during pharmacotherapy.

In line to what is being discussed, MEs are also commonly reported in patients with T2DM (10, 16). Among such patients, polypharmacy contributes as a major risk factor resulting in the development of MEs and drug related problems (17, 18). The risk of polypharmacy as a potential contributor to MEs also increases when medicines are prescribed within the hospital settings with short- and long-term treatment regimens. Therefore, knowledge regarding the quality use of medicine in accordance to established treatment guidelines (proposed by NICE, FDA etc.) is a key factor in minimizing MEs in chronic diseases (19). Within this context, Roelens et al. in their conceptual framework model claimed that quality prescribing is determined by the ability to apply explicit medication-related knowledge (20). The authors also stated that satisfactory level of knowledge is the starting point for quality use of medicine which guarantees optimal pharmaceutical care with least probability of developing MEs (20). This is also linked with the understanding of polypharmacy and the associated errors which is of prime importance especially for the prescribers to avoid any negative experience in clinical settings (21).

Shifting our concerns to quality use of medicines in developing countries, physicians are the principal source of information for the patients. Other sources (pharmacists, patient information leaflets, drug information centers and the internet) are least accessible because of non-availability or affordability for majority of the population. In addition, poor literacy (both formal and health) rate is another factor that hinders the accessibility of quality information (22). Focusing MEs in Pakistan, although there is no official data reported from the country, MEs are reported to cause half a million deaths in Pakistan (23). Riaz et al. in their review claimed that while MEs occur every day, healthcare professionals are not fully aware of the damages done by MEs in terms of patients' discomfort and economic burden. Also, the authors recommended providing information about MEs the healthcare providers so to avoid complications in clinical settings (24). Even though much is reported in literature regarding MEs, prescribers' perception of MEs is least reported and there is scarcity of information from Pakistan. We strongly believe that to design a targeted intervention for reducing MEs, it is vital to identify the prescribers' discernment toward MEs. Such perceptions and notions will be helpful in identifying the gaps between “what is known and what is to be achieved.” Consequently, we designed this study because of 2-fold reasons. Firstly, the said perception is not reported in literature from Pakistan and secondly, we also aimed to assess the perception qualitatively to get rich and in-depth information that will be further utilized in developing quantitative research. For that reason, the current study is aimed to qualitatively highlight physicians' perceptions, experiences, and expectations on MEs while managing T2DM patients is a public healthcare institute of Quetta city, Pakistan.

## Methods

### Study Design and Settings

We adopted a qualitative study design because of flexibility in approach and to highlight a detailed exploration of respondents' attitudes, experiences, and intentions (25, 26). Qualitative methods generate a wide range of ideas and opinions, as well as divulge viewpoint and differences among groups. In addition, with topics lacking baseline information, qualitative methods generate a blueprint that helps in designing a roadmap for future studies (27). For that reason, we believed that using qualitative methods for this study were unmatched choice for inductive approaches aimed at generating concepts which have far more potential for research than any other models (28).

The study was conducted at Sandeman Provincial Hospital Quetta. Sandeman Provincial Hospital is a tertiary care, teaching hospital and being public in nature is approached by majority of the population (29).

### Study Participants, Criteria, and Sampling

All medical practitioners registered by the Pakistan Medical Commission and practicing at the medical wards were approached for the study. The medicine ward provides inpatients facilities free of cost. Furthermore, patients with complication of T2DM are referred to the medicine wards and are managed there. Based on the study objectives, it was apparent to adopt the purposive sampling method (30). Physicians on house jobs were excluded from the study as drug therapy is initiated by senior physicians in the wards.

### The Interview Guide (Peer Review, and Pilot Study)

The research team constructed a semi-structured interview guide after an extensive literature review (31–35). The guide was established with widely framed, open-ended questions that gave enough freedom to the physicians for explaining their viewpoints. Parallel, the respondents were also encouraged to provide their own narratives and to share further information relevant to MEs during clinical encounters.

The guide was constructed in the English language was subjected to peer review assessment through a panel of experts (two Professors in Medicine). Once the dependability was ensured, the guide was piloted with physicians to ensure that topics to be discussed were at the level that respondents would comprehend with ease. The preliminary data and conclusion confirmed that the discussion topics were enough and appropriately phrased to answer research questions and to minimize connectivity threats. As the dependability of the discussion guide was ensured, it was made available for the main study. Data, members of the expert panel and participants of the pilot study were not involved in the final analysis.

### Interview Procedure, Data Collection and Analysis

The first author conducted the interviews in the offices of the medicine wards. All interviews were conducted in English and participants were briefed about the study objectives before the interviews. A debriefing session was again conducted at the end of the discussion. The interviews started with an ice-breaking session. Probing questions were asked in between conversations to clarify the meanings of responses and to gain insight of the topic being discussed.

Each interview was audio-recorded that lasted for approximately half an hour. To draw in-depth views, the freedom to express additional reviews and comments was given to the physicians. The second author acted as an observer while the third author assisted in monitoring the field notes, facial expressions and body language that complemented the audio recordings. Interviews were conducted until thematic saturation was reached (36, 37). By using the phenomenology-based approach, the research team analyzed the recordings (verbatim) and later arranged an informal gathering where physicians were presented with the finalized interview scripts (38). Physicians were asked for confirmation of precision and accuracy of words, ideas, and jargon used during the script analysis. NVivo® was used for coding and analysis through iterations (39) and inconsistencies were resolved through mutual consensus. We used the inductive approach for identification and extraction of the themes. The coding framework clarified the themes and ensured the significance. All emerging themes and subthemes were discussed among the research team for accuracy and were presented for data inference and interpretation. Based on the analysis, a descriptive text was drafted, producing a dialog between the identified themes and the inferences drawn by this study.

### Ethical Approval

Institutional review board approved the study protocol [UoB/Reg:/GSO/67]. Written consent was taken from the respondents before the interviews. The physicians were introduced to the nature of the research prior to the beginning of the interviews, were made secure of the confidentiality of their responses and their right to withdraw from the study.

## Results

### Demographic Characteristics of Study Respondents

Although the saturation was reached at the 13th interview, we conducted additional two interviews to ensure the saturation. Consequently, 15 physicians were interviewed out of which 73% were males and the highest qualification was Fellowship of the College of Physicians and Surgeons (Table 1).

**Table 1:** Demographic characteristics of the physicians.

**Characteristics**	***N***	**%**
***Age group (years)***		
25–35	7	46.7
36–45	3	20
45–55	5	33.4
***Gender***		
Male	11	73.4
Female	04	26.6
***Practicing experience (years)***		
1–10	10	66.7
>10	05	33.3
***Current position***		
Consultant	04	26.6
Registrar	05	33.3
Postgraduate	06	40.1
***Highest qualification***		
Fellowship of the College of Physicians and Surgeons	04	26.6
Fellowship of the College of Physicians and Surgeons (part one)	09	60.1
Bachelor of Medicine and Bachelor of Surgery	2	13.3

**Figure 1:**
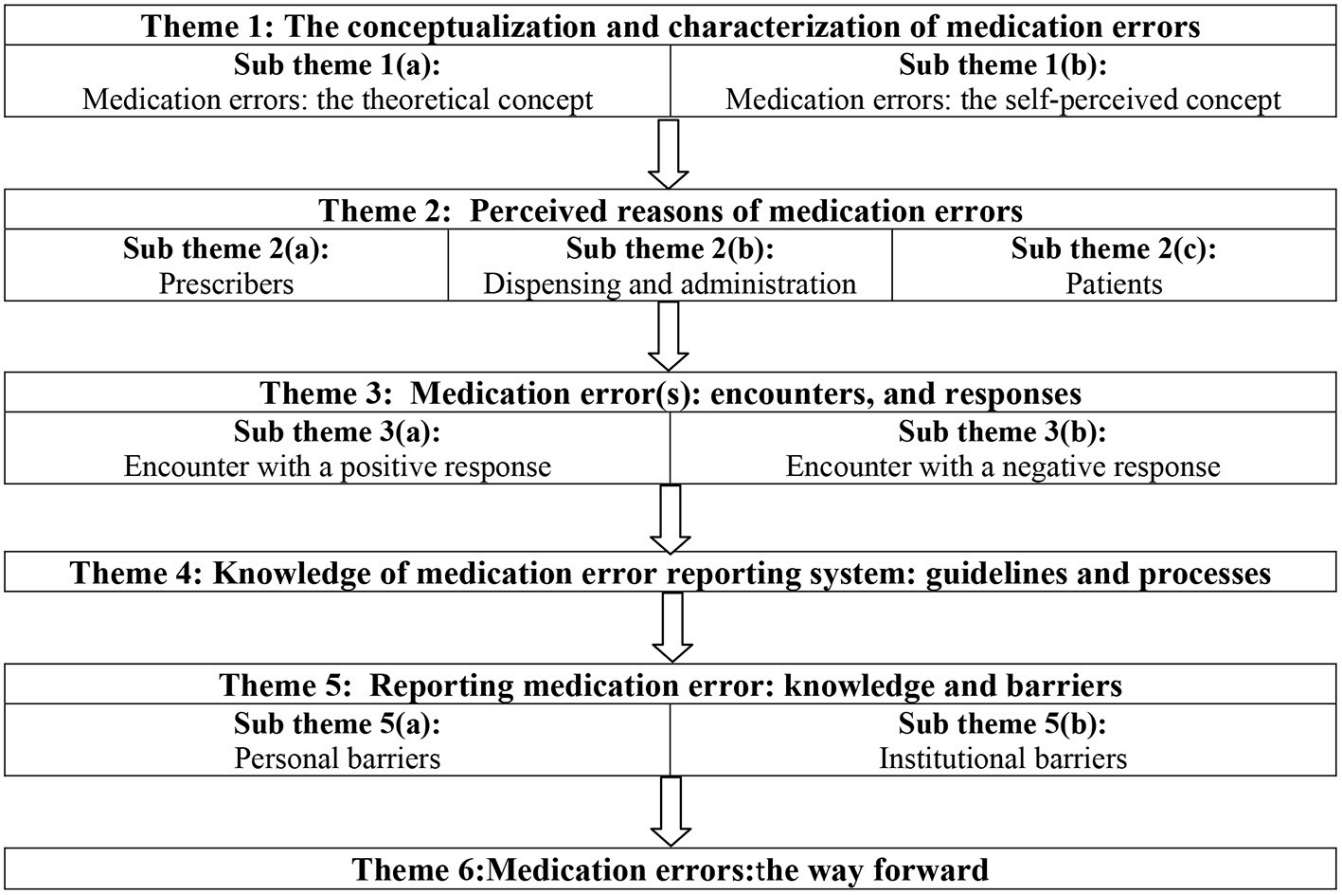
Schematic presentation of themes and sub themes identified during data analysis.

Thematic content analysis resulted in the following themes and subthemes (Figure 1).

#### Theme 1: The Conceptualization and Characterization of Medication Errors

According to van Mil et al., “*not all MEs errors are a problem for patient outcomes and not all medicine-related patient problems are caused by MEs*” (9). We must remember that MEs can be manifest, and potential. Thus, it is imperative that physicians should have a comprehensive understanding of MEs and its related philosophies so to avoid obnoxious events in the clinical settings. Our study highlighted that physician had mixed conceptualization and characterization of MEs that are discussed subsequently.

##### Subtheme 1[a]: Defining Medication Errors: The Theoretical Concept

The term MEs were known to the physicians, but majority of the respondents were unaware of the theoretical concept and philosophy. Limited information was extracted when the respondents were asked about the definition of MEs according to established guidelines.

***“Yes, I know about MEs. Defining MEs (hmmm), I am sorry I am not well versed” [Physician 5]***

Besides, another physician (physician 2) admitted his poor knowledge toward MEs and explained the reasons of the ignorance.

***“I do confront MEs in my practice but in terms of defining the ideology, I admit its lacking. For that I need to go to the basics, and I do not have time for that” [Physician 2]***

On the contrary, three physicians [Physician 1, 7 and 11] knew the theoretical definition of MEs, the philosophy, potential harms, and the agencies that regulate medication errors around the globe.

***“Yes, I am aware of what MEs are and I keep myself updated regarding MEs and the current global scenario.” [Physician 7]***

##### Subtheme 1 [b]: Medication Errors: The Self-Perceived Concept

Interestingly, for majority of the physicians the concept of MEs was unclear. According to the transcripts and discussions during the interview sessions, our respondents were of opinion that all medicines have the tendency to produce adverse effects and hence errors are inevitable. Few of our respondents believed that such errors are avoidable (to some extent) but in general MEs are non-preventable. Physicians related this belief to the number of stakeholders involved in the medicine management system that is beyond the reach of the healthcare professionals (physicians).

***“Medication errors are likely to occur, and it is impossible to avoid such events. Even if we prescribe and dispense the right medication, the patients have the tendency to misuse, and this is beyond our capacity.” [Physician 8]***

While some of our respondents had a positive perception toward preventable MEs, they showed certain reservation toward the ambiguities of the healthcare system and the societal factors. Such factors were identified to play a key role in the management of medication errors.

***“I do agree that MEs are preventable events. But what about patient-physician relationships, miscommunications, deprived healthcare, and poor health literacy in our society? Even if we play our part in prescribing, MEs can go off any time and unfortunately we cannot do anything about it.” [Physician 12]***

Positively, two physicians were confident that if the healthcare professionals and the patients collaborate, there are chances that MEs are preventable.

***“A collaborative effort from the healthcare professionals and the patients is required. If we can educate the patients/caregivers about quality use of medicines, we can reduce the frequency of MEs. Nevertheless, it is not going to be easy and sincere efforts are needed to reduce events leading to MEs.” [Physician 1***

#### Theme 2: Perceived Reasons of Medication Errors

Helper and Strand while defining pharmaceutical care emphasized on responsible provision of drug therapy. However, they also acknowledged the hazards because of MEs (40). Agreeing with this thought, Montesi and Lechi emphasized that identification of the reasons and early MEs detection encourages quality use of medications in a healthcare system (41). Nevertheless, this is only possible when the healthcare practitioners are well-versed with the predictors of MEs. Correlating, the literature does report a number of reasons that results in MEs (42) and for that reason we wanted to inquire how equipped our physicians are when it came to the reasons of MEs in their clinical settings.

Physicians of this study were quite open in answering and identifying the reasons of MEs at their practice site. Where physicians highlighted dispensing and patient related factors, they had no hesitancy in admitting themselves as a contributing factor toward MEs. Other than this, some other factors were also highlighted and that are discussed subsequently.

##### Subtheme 2[a]: Prescribers

According to one of the respondents (physician 9), “***we have many duties in a hospital. Plus, the patient flow in this hospital is increasing day by day. In such rush, a lapse ofjudgment is common, and a medication error can occur***”. Adding to the statement, disruptions and disturbances were also highlighted as a reason of medication errors.

***“ This hospital is approached by the poor. On average and in working hours, I usually interact with 60 patients every day. With such huge numbers, distractions are possible and can result in MEs because of handwriting, wrong dose, inappropriate drug selection etc.” [Physician 11]***

##### Subtheme 2[b]: Dispensing and Administration

Both dispensing and administration of medicines are long known as a high-risk factor of MEs. Similarly, this was acknowledged by our respondents where widely held perception of MEs was because of incorrect dispensing or administration of medications by the pharmacists or the paramedics. Assuredly, almost all physicians were of a strong opinion that majority of MEs occur during the dispensing and administration phase and less in the prescribing phase.

***“Majority of our (prescribers) prescriptions are accurate but are either inappropriately dispensed or irrationally administered to the patients. Therefore, MEs are more of a dispensing/administrative problem and not a prescribing one.” [Physician 5]***

##### Subtheme 2[c]: Patients

According to Sears et al., MEs are more likely to occur four times more often in the community when compared to the hospital (43). The same was also recognized and highlighted by the study respondents. Yet again, majority of the physicians agreed that MEs are on the part of the patients, and they have no control over the patients when they are using medications.

***“The patients are the reasons why MEs occur. They self-medicate, use multiple medications and this results in MEs. Unfortunately, this is beyond our reach and there is nothing we can do about it.” [Physician 4]***

#### Theme 3: Medication Error[s]: Encounters, and Responses

The way in which physicians respond to MEs can turn their experiences into powerful opportunities of learning. This also makes sure that MEs are recognized and will not be repeated. Therefore, it was obvious to get information about physicians' encounters of MEs and their response to the error in return.

##### Subtheme 3[a]: Encounters With a Positive Response

While managing T2DM patients with comorbidities, there are high chances of MEs. Chances of MEs are augmented when in-patients are considered and maintenance of the vital signs such as blood glucose level and blood pressure is fundamental. All study respondents had encounter MEs during their practice and majority of them were confident that they responded to the error in a positive and professional manner. Although MEs should be inevitable, it was encouraging to know that the physicians were able to respond accordingly.

***“A diabetic patient with serious hyperglycemia was brought to the ward. On discharge, I came to know that the patient was making insulin made dosage error and his measured dose differed from the prescribed dosage. This was a potentially serious medication error and thus I have to intervene and readjust the dosage.” [Physician 8]***

##### Subtheme 3[b]: Encounters With a Negative Response

Physician 10 clarified that ***“when diabetic patients are admitted to my ward, the reasons of the admission are repeatedly unknown, and physicians have to manage based on the signs and symptoms. Often at time, the conditions become worse, and we are unable to response because of no available information.”*** Additionally, the very respondent also recalled his experience where a patient took multiple medications, was brought unconscious to the hospital. The physicians were unable to respond and hence resulted in the death of the patient.

#### Theme 4: Knowledge of Medication Error Reporting System: Guidelines and Processes

The objectives of following guidelines for the management of T2DM are to enhance appositeness of clinical practice. When guidelines are followed, quality of diabetic care is improved that leads to better patient outcomes. Correlating, Woolf et al. commented that guidelines improve the consistency of care that ensures patients are cared in the same manner regardless of where or by whom they are treated (44). Concerning the use and implementation of guidelines in clinical practice, physicians of the current study were of the same opinion. The sliding scale was used as a standard for the management of T2DM in their wards. Other than that, majority of the respondents were unaware of the current guidelines that are developed and being implemented in the management of T2DM.

***“Seldomly, I follow the guidelines. However, I do use the sliding scale readings and adjust units accordingly.” [Respondent 02]***

During the informal discussion, we observed mixed responses regarding the sliding scale itself. Where few of the physicians did not consider sliding scale as a guideline, some of them answered that because they are unaware of the guidelines hence must follow sliding scale. Nevertheless, only one respondent claimed of having knowledge of new guideline and have attended trainings and workshops to manage T2DM patients.

***“I am aware of the ADA and NICE guidelines. I use the recommendations during T2DM management in my ward.” [Physician 7]***

It is now acknowledged that guidelines and the applicability provide better results worldwide. Guidelines also contribute to long term benefit to the healthcare system and patients. Interestingly, when asked about benefits of using guideline, three of our respondents believed that guidelines are beneficial but to some extent. They believed together with guidelines feedback from the experience physicians is also important.

***“Guidelines are important and provide benefit. But the input of other experienced physicians is what matter most.” [Respondent 13]***

#### Theme 5: Reporting Medication Error: Knowledge and Barriers

Medication errors are expected in a clinical setup, however; timely and appropriate reporting of the errors minimizes the chances of recurrence. Therefore, in addition to an appropriate error reporting system, it is also essential that the physicians are aware of the medication error reporting process. Although physicians of the current study were aware of medication error reporting system, as expected, poor understanding of MEs, importance of reporting MEs, lack of a reporting system, work environment/culture and certain other personal factors were rated as barriers in reporting MEs. Consequently, two sub themes were identified during data abstraction i.e., personal, and institutional barriers in reporting medication errors.

##### Subtheme 5[a]: Personal Barriers

***“Reporting system! Yes, I know about the reporting mechanism but its time consuming. Furthermore, I am not sure that it is the job of the physicians to report MEs.” [Physician 4]***

##### Subtheme 5[b]: Institutional Barriers

***“I am a strong advocate of reporting MEs to the authorities. However, the hospital does not have a reporting system in practice. Additionally, we are short of other resources too. So, at present this is not possible in our hospital.” [Physician 9]***

#### Theme 6: Medication Errors: The Way Forward

In a nutshell, although physicians of the current study had mixed responses and attitudes toward medication safety and its correlates, they were quite positive in establishing a system of medication error reporting. They strongly believed that prevention of MEs and addressing them is an important task, and all stakeholders need to come forward to address this issue. To get a clearer picture, we have summarized the responses in Table 2 that presents the opinions and recommendation of the physicians.

**Table 2:** Physicians' opinions and recommendations toward medication safety.

**S. No**	**Opinions/recommendations**
1.	Physicians need training and sessions on guidelines and reporting mechanisms.
2.	Interventions are needed (both tailored and generalized) to avoid medication errors.
3.	There should be an independent section designated to address medication errors in hospital.
4.	Pharmacists must be trained to handle medication errors alongside with the physicians.
5.	Paramedics must be continuously trained and supervised by the medication error reporting committee to minimize medication errors.

## Discussion

Although healthcare professionals undergo extensive medical training during their studies, they need to continuously update their knowledge during time. Therefore, developed countries emphasis more on continuous medical education when compared to developing countries. Societies around the globe demand that a physician must be error free and that is achievable through occupational agility, and responsibility toward patient care (45). While medical education equips the physicians with technical and scientific knowledge but mature, and balanced personality capable of understanding biopsychosocial construct of the patient is desirable. This is the reason why American Medical Association recommended to transform medical education and emphasized more on improving patient safety, healthcare system knowledge, care-based learning, and quality of medical attention (46). This is only possible when the physicians are continuously updated with new technologies and happenings in the healthcare domain around the globe. This also make sure that the physicians are equipped with up-to-date information and procedures while managing patients at both clinical and non-clinical healthcare settings.

Relating to continuous medical education (CME) and developing countries, once the physicians qualify for registration, they are licensed to practice for life. Unlike developed world there is no legislation to bound physicians for mandatory CME for extension of their practicing license (47). Therefore, with swift and rapid changes of guidelines, procedures, and management protocols, physicians of the developing world obviously lack updated information. The situation goes off hands when chronic diseases are taken into consideration. Furthermore, the management of chronic diseases in low-resource health settings are also compromised by the socio-economic situation. All of the above mentioned factors results in miscalculations, underestimations and hence MEs are likely to happen due to complex treatment regimens and use of multiple medications (48).

Results of the current study highlighted that physician had mixed conceptualization and characterization of MEs. Even though MEs were known to the physicians, majority of the respondents were unaware of the theoretical concept and philosophy. Similarly, a study conducted in Brazil reported that physicians considered MEs as a tool to confirm what theory talks about this topic (49). Similarly, where physicians are engaged in managing a lot of patients, they experience a paradox to conceptualize the concept of MEs. Interestingly, for majority of the physicians, the concept of MEs was unclear. The respondents were of opinion that all medicines have the tendency to produce adverse effects and hence errors are inevitable. Our findings are parallel to what is reported by Kapaki et al. The authors highlighted that healthcare professionals often oversight MEs during planning and execution of healthcare provision, and that contributes to impairment of patient's health in particular and health system in general (50).

Our study respondents when asked about MEs felt no hesitancy in admitting themselves as a contributing factor toward MEs. The apparent reasons mentioned in our result (Theme-2a) are supported by Shanafelt et al., where 76% of the physicians suffered from professional burnout and they were more likely to report inappropriate healthcare practices for instance omission in diagnostic treatment, inappropriate behavior toward patients, and MEs on weekly basis (51). Assuredly, almost all physicians were of a strong opinion that majority of MEs occur during the dispensing and administration phase and less in the prescribing phase. The Malaysian study conducted by Chua et al., revealed that drug administration errors were common in the Malaysian hospitals and the error rate was 11.4% (52). The staff responsible for preparation and administration of medications are susceptible to make unsafe actions and their errors are strongly affected by local working conditions. Yet again, majority of the physicians agreed that MEs are on the part of the patients, and they have no control over the patients when they are using medications. Breuker et al., also described the identical concept in diabetic patients with different comorbidities and polypharmacy are at high risk of MEs (53).

Our study respondents did encounter MEs during their practice and majority of them were confident that they responded to the error in a positive and professional manner. Thomas et al. also found comparable results where most of the physicians used to worry about MEs fearing that an error might harm patient. In addition, physicians also feared of diminished self-confidence, and loosing respect of colleagues (54). Most of the respondents were unaware of the current guidelines that are developed and being implemented in the management of DM. Likewise it was also emphasized that physicians lack confidence in their knowledge of guidelines and expertise in a specific task like enabling patient behavior change and initiating insulin (55). This is true because clinicians are faced with various challenges in complex management of diabetes. They strive to meet evolving management targets within defined time and resources, and express dissatisfaction with resulting compromises in care.

While physicians of the current study were aware of MEs reporting system, poor understanding of MEs, lack of a reporting system, work environment / culture and certain other factors were rated as barriers in reporting MEs. Soydemir et al., also revealed that physicians were reluctant to report MEs because they were afraid of being blamed, losing their status, and legal sanctions. The literature has exposed that administrative attitudes effect the error reporting directly, in a way that every negative response will negatively affect error-reporting rates (56). In other studies, physicians highlighted that they do not practice error reporting because there is lack of error reporting system, it is a time-taking process and there are deficiencies in the system. The key barriers identified were insufficiency of information regarding reporting system, the paucity of resources to educate about reporting, and mistrust in reporting system (57, 58).

In line to what has been discussed, physicians believed that prevention of MEs and addressing them is important and must be addressed. They recommended training sessions on guidelines and reporting system, implementation of interventions to avoid MEs, establishment of independent section to address MEs, involvement of pharmacists, and strict supervision of paramedics to minimize MEs. Literature has also reported comparable suggestions where physicians believed that interventions would be very effective to decrease MEs (59), requiring hospitals to develop systems for avoiding MEs (60), pharmacists to help physicians in reducing MEs (61), and training of in-service nurses (62). Medication error reporting system in Pakistan often remain unnoticed and these errors can be minimized by implementing strategies like electronic prescribing, computerized physician order entry, bar coding of drug labels and use of an effective MEs (24).

## Conclusion

With a longer life expectancy and growing population, the frequent incidences of MEs are expected to increase. Physicians of the current study had mixed abstraction of MEs. Consequently, efforts should be made to ameliorate overall physicians' communication and transformation of care. Possible strategies to overcome this problem include review of admission and discharge reconciliation, encourage postgraduate trainee to question indication and utility of medication, team rounding with a pharmacist, and disseminating data regarding MEs. The only way to prevent medication errors is by creating culture of teamwork, communication and to learn from our mistakes.

## Strengths and Limitations

Although MEs are frequently reported in literature, physicians' perceptions, experiences, and recommendations for the prevention of MEs are least reported form Pakistan. Therefore, the current study is pioneer study from this part of the world exploring in-depth view of MEs from physicians' point of view. Nevertheless, this qualitative study was conducted in one hospital of the city which is not representative the issue of generalizability is always questionable.

## Recommendations

Based on the generalizability of qualitative studies, a comprehensive study is recommended throughout the country to generalize the results. This is approachable by conducting similar studies in other parts of the country and later designing a nationwide quantitative study.

## Data Availability Statement

The original contributions presented in the study are included in the article/supplementary material, further inquiries can be directed to the corresponding author.

## Ethics Statement

The studies involving human participants were reviewed and approved by Faculty of Pharmacy and Health Sciences, University of Baluchistan, Quetta, Pakistan. The patients/participants provided their written informed consent to participate in this study.

## Author Contributions

QI, FS, and AK conceptualized the study and developed the interview guide. MH, MK, AS, and AR conducted the interview and compiled the information while SH, NA, and MA analyzed and interpreted the data. ZI and FK were involved in the initial write-up and review. The study was supervised by FS, NA, and AK. All authors have met the criteria for authorship, had a role in preparing the manuscript, and approved the final manuscript.

## Conflict of Interest

The authors declare that the research was conducted in the absence of any commercial or financial relationships that could be construed as a potential conflict of interest.

## Publisher's Note

All claims expressed in this article are solely those of the authors and do not necessarily represent those of their affiliated organizations, or those of the publisher, the editors and the reviewers. Any product that may be evaluated in this article, or claim that may be made by its manufacturer, is not guaranteed or endorsed by the publisher.
